# Cellular senescence contributes to mechanical ventilation-induced diaphragm dysfunction by upregulating p53 signalling pathways

**DOI:** 10.1186/s12890-023-02662-7

**Published:** 2023-12-14

**Authors:** Weimin Shen, Ye Jiang, Ying Xu, Xiaoli Qian, Jianwei Jia, Yuejia Ding, Yuhan He, Qing Pan, Jinyang Zhuang, Huiqing Ge, Peifeng Xu

**Affiliations:** 1 Department of Respiratory Care, Regional Medical Center for National Institute of Respiratory Diseases, Sir Run Run Shaw Hospital, School of Medicine, Zhejiang University, Qingchun East Rd. 3, Hangzhou, 310016 China; 2 College of Information Engineering, Zhejiang University of Technology, Hangzhou, 310023 China; 3 Huashan Hospital affiliated to Fudan University, Shanghai, 200040 China

**Keywords:** Mechanical ventilation, Diaphragm dysfunction, Cellular senescence, p53-p21 axis

## Abstract

**Background:**

Mechanical ventilation can cause acute atrophy and injury in the diaphragm, which are related to adverse clinical results. However, the underlying mechanisms of ventilation-induced diaphragm dysfunction (VIDD) have not been well elucidated. The current study aimed to explore the role of cellular senescence in VIDD.

**Methods:**

A total of twelve New Zealand rabbits were randomly divided into 2 groups: (1) spontaneously breathing anaesthetized animals (the CON group) and (2) mechanically ventilated animals (for 48 h) in V-ACV mode (the MV group). Respiratory parameters were collected during ventilation. Diaphragm were collected for further analyses.

**Results:**

Compared to those in the CON group, the percentage and density of sarcomere disruption in the MV group were much higher (*p* < 0.001, both). The mRNA expression of MAFbx and MuRF1 was upregulated in the MV group (*p* = 0.003 and *p* = 0.006, respectively). Compared to that in the CON group, the expression of MAFbx and MuRF1 detected by western blotting was also upregulated (*p* = 0.02 and *p* = 0.03, respectively). Moreover, RNA-seq showed that genes associated with senescence were remarkably enriched in the MV group. The mRNA expression of related genes was further verified by q-PCR (Pai1: *p* = 0.009; MMP9: *p* = 0.008). Transverse cross-sections of diaphragm myofibrils in the MV group showed more intensive positive staining of SA-βGal than those in the CON group. p53-p21 axis signalling was elevated in the MV group. The mRNA expression of p53 and p21 was significantly upregulated (*p* = 0.02 and *p* = 0.05, respectively). The western blot results also showed upregulation of p53 and p21 protein expression (*p* = 0.03 and *p* = 0.05, respectively). Moreover, the p21-positive staining in immunofluorescence and immunohistochemistry in the MV group was much more intense than that in the CON group (*p* < 0.001, both).

**Conclusions:**

In a rabbit model, we demonstrated that mechanical ventilation in A/C mode for 48 h can still significantly induce ultrastructural damage and atrophy of the diaphragm. Moreover, p53-dependent senescence might play a role in mechanical ventilation-induced dysfunction. These findings might provide novel therapeutic targets for VIDD.

**Supplementary Information:**

The online version contains supplementary material available at 10.1186/s12890-023-02662-7.

## Introduction

Mechanical ventilation is widely adopted as a medical procedure that can be a life-saving supportive therapy for acute respiratory failure patients. However, several studies have established that prolonged mechanical ventilation can lead to a complication termed ventilation-induced diaphragm dysfunction (VIDD), making it difficult for patients to wean from the ventilator [1, 2]. Specifically, inappropriate mechanical ventilation leads to diaphragm muscle atrophy and weakness.

Our understanding of VIDD has grown rapidly over the past few decades. Since it was first suggested that prolonged mechanical ventilation contributes to infant diaphragm dysfunction in 1988 [3], numerous studies on animal and human models have confirmed that controlled mechanical ventilation (CMV) not only leads to impaired force-generating capacity but also induces muscle atrophy and structural injury in the diaphragm [4–7]. In previous studies, findings have shown that an imbalance in diaphragm protein homeostasis is the underlying molecular mechanism of VIDD, which corresponds to decreased protein synthesis and increased protein degradation [6, 8, 9]. The activation of the ubiquitin proteasome system (UPS) induced by mitochondrial oxidative stress (MOS) is considered to play a potent role in diaphragm protein degradation and atrophy [10, 11]. Additionally, other molecular events have also been shown to be involved in the pathogenesis of VIDD, such as autophagy, apoptosis, and calpain activity [5, 12–14].

Most previous studies on VIDD have focused on the controlled mechanical ventilation (CMV) mode. In this mode, animals are under deep anesthesia, which can help eliminate the diaphragmatic electrical activity to reveal the mechanism of VIDD [15, 16]. Moreover, this ventilator-induced diaphragmatic dysfunction is significantly correlated with the duration of mechanical ventilation [17]. Therefore, compared with the short-term mechanical ventilation model (less than 24 h), the establishment of a long-term model will be more in line with clinical practice for the study of diaphragmatic dysfunction. In addition, previous studies have confirmed that, compared with CMV, assisted-controlled ventilation (ACV) retains the nerve stimulation and contractile activity of the diaphragm, thus exerting a certain protective effect on the diaphragm [18]. However, this protective effect can only alleviate diaphragm atrophy to a certain extent. In fact, diaphragmatic dysfunction is still difficult to avoid after prolonged exposure to ACV mode. As the most commonly used mode of mechanical ventilation in intensive care units, attention to the mechanism of diaphragmatic dysfunction during long-term ACV is expected to provide new ideas for preventing VIDD and improving clinical outcomes of patients [19]. Therefore, in our current study, we focus on the effect of long-term continuous ACV on the diaphragm, which may more consistent with clinical practice. In this study, we found that cellular senescence is a novel molecular event contributing to the development of VIDD in the ACV mode for 48 h.

Cellular senescence is defined as a permanent arrest of the cell cycle that mainly manifests with increased activity of senescence-related β-galactosidase, the involvement of multiple cyclin-dependent kinase inhibitors (CDKi), increased secretion of proinflammatory cytokines and other features [20]. It enhances tissue/organ remodelling in the process of development and injury repair but can also cause decreased regenerative potential and tissue function, leading to inflammation and tumorigenesis in aged organisms [20]. In muscle repair, senescence plays both beneficial and detrimental roles [21–23]. On the one hand, the senescence-associated secretory phenotype (SASP) enables skeletal muscle reprogramming and promotes stemness and cell plasticity [21, 24, 25]. On the other hand, p53‐dependent persistent senescence is pernicious during the process of muscle repair [23]. As one of the most well-established downstream targets of p53, p21 is crucial for p53-dependent cell cycle arrest mediation. Stress-induced p53-dependent induction of p21 inhibits CDK2-mediated RB phosphorylation to promote cell cycle arrest [26]. Other studies have also indicated that upregulated p53 and p21 protein levels contribute to the diaphragm dysfunction induced by cigarette smoke exposure [27].

In this context, the current study aimed to determine the role of p53 signalling pathways in inducing cellular senescence in the diaphragm during mechanical ventilation. The findings may help elucidate the cause of VIDD.

## Materials and methods

### Study design

The research was conducted with the approval of the Animal Experiments Committee of the Zhejiang University School of Medicine, Zhejiang, China. A total of twelve New Zealand white rabbits (six per group) weighing 2.0 to 2.5 kg were studied. All animals were randomly divided into two groups.

Animals were under general anaesthesia with 35 mg/kg sodium pentobarbital by intraperitoneal injection. After reaching the surgical anaesthesia level, the animals were weighed and intubated through tracheostomy. Two No. 20 catheters were placed in two ear marginal veins for liquid and drug administration. One of the catheters was used for normal saline (0.9% normal saline), and the other catheter was used for intravenous dexmedetomidine (0.2–0.7 µg/kg/h) delivery by a micropump. Mild sedation was provided, and the drinking reflex was protected to ensure consistency with clinical conditions and reduce the respiratory effect of sedatives. Arterial blood gas parameters were collected after 48 h. The experimental ventilator was an SV800 ventilator (neonatal type: Mindray, Shenzhen, China) with a neonate circuit. The ventilator settings were as follows: volume control mode, tidal volume 8ml/kg, respiratory rate 40–50 bpm, and PEEP level of 0 cmH_2_O. During the experiment, the pressure and airflow waveforms of the ventilator were collected through the RS-232 output port of the ventilator, and the respiratory parameters were continuously recorded (Respcare™, Hangzhou ZhiRuiSi, China). All operations were performed using aseptic techniques. Each rabbit was insulated with a heated electric blanket. The experimental duration was 2 days. For animals receiving mechanical ventilation for 48 h, a feeding tube was inserted into the stomach through an incision in the oesophagus.

After 48 h, the animals were euthanized with an overdose of sodium pentobarbital, and the diaphragm muscles were carefully dissected. The diaphragm specimens were divided into three parts: one was treated with 2.5% glutaraldehyde for ultrastructural observation, another was immersed in 4% paraformaldehyde at 4 °C overnight for paraffin embedding, and the third was immediately frozen in liquid nitrogen and stored at -80 °C.

### Ultrastructural observations

Tissues of the diaphragm were cut into 1-1.5 mm^3^ slices and then fixed in 2.5% glutaraldehyde at 4 °C for 12 h. Then, the samples were fixed, dehydrated, dried and sectioned according to standard methods. Eventually, the ultrastructure of the diaphragmatic sarcomeres was observed under a transmission electron microscope (TEM) (JEM-1230; JEOL Company, Japan), and photomicrographs were taken. Morphological analysis was performed at a magnification of ×10,000, and five fields of view were randomly selected from each sample for statistical analysis. To quantify the injury, the density (n/100 µm^2^) and proportion (n/total number of sarcomeres) of disrupted sarcomeres were normalized to the micrographic area.

### SA-βGal staining

First, quick-frozen diaphragm muscle samples were cut into 20-µm cross-sections. An appropriate volume of β-galactosidase staining fixative was added to every section to fix the tissues. Then, tissues were incubated overnight at 37 °C in an SA-βGal staining solution (C0602, Beyotime Inc., China). Under an ordinary optical microscope (Olympus BX61, Tokyo, Japan), the blue-stained cells were senescent cells.

### Immunofluorescence (IF)

The diaphragm tissue was fixed in 4% paraformaldehyde at 4 °C for 24 h and then embedded in paraffin. Eight-micrometer cross-sections were prepared and incubated with a primary antibody against p21 (ab107099, 1:200, Abcam, Britain) at 4 °C overnight. The washed tissues were incubated with mixtures of secondary antibody (A-11,081, 1:500, Invitrogen, United States), wheat germ agglutinin (W11261, 1:1000, Invitrogen, United States) and Hoechst (H1399, 1:500, Invitrogen, United States) for 60 min and then observed under an Olympus microscope (Olympus IX83-FV3000-OSR, Tokyo, Japan).

### Immunohistochemistry (IHC)

The sample is prepared as described above. The tissue slides were deparaffinized by xylene, and rehydrated by Gradient concentration of alcohol. After rinsing in distilled water, 3% H_2_O_2_ solution was utilized to block endogenous peroxidase activity of tissue slide. The tissues slides were then incubated with a primary antibody against p21 (10355-1-AP, 1:200, proteintech, China) overnight at 4 °C. The washed tissues were incubated with secondary antibody (ab205718, 1:1000, Abcam, Britain) at 4 °C for 60 min. The hematoxylin was utilized to counterstain tissue slides for 2 min and then tissue slides were observed under microscope (Olympus VS200, Tokyo, Japan).

### Quantitative RT‒qPCR

Total RNA from diaphragm tissues was extracted by an AxyPrep Multisource Total RNA Maxiprep Kit (AP-MX-MS-RNA-10G, Axygen, United States) and reverse-transcribed into cDNA using the HiScript® II Reverse Transcriptase Kit (R223, Vazyme, China). Real-time PCR was carried out by applying ChamQ Universal SYBR qPCR Master Mix (Q711, Vazyme, China) following the manufacturer’s protocol. For each sample, the 2^−∆∆Ct^ method was used to evaluate the mRNA abundance for target genes, and GAPDH was adopted as the housekeeping gene. The respective primers are shown in Table 1.

**Table 1:** Primers used in Quantitative RT-qPCR study

Gene	Forward Primer	Reverse Primer
ANKRD1	CGCCCGAGATAAGTTGCTCA	TTCTCGGTCTTTGGCGTTCA
CHEK2	GGACCCAAAGGTGCGTCTTA	TGTGGCCTCAGCATTCTCTG
GAPDH	TAAGAGCCCTCAAACCACCG	AAGAGGGGCAGATTCTCAGC
MAFbx	CCACGCTCTCGTTTCAGACT	CCAAGGGGGCAACTCATTCT
MMP9	GTGTGAGTACCCGGAACGAG	TGTATCCGGCAAACTGGTCC
MuRF1	TGCTCAGAGAGCAGGGAGTA	TTCCAATCCGGGTTCAGTGG
p21	GAAGGGCGTGGATAGCACTT	GTGCCCATCTCAGAGTCACC
p53	CTCCTGAGGACTGGCGAC	AGCCAGTTTGCAACGTCTTC
Pai1	CCACGGATGCCATCTTCGT	AAAGTCCACCTGCTTGACCG

### Western blot analysis

The diaphragm protein lysates were separated by SDS‒PAGE and then transferred to a polyvinylidene fluoride membrane (IPVH00010, Millipore, United States). The membranes were clipped according to target proteins molecular weight position on the instruction of the protein marker. After that, the membrane was blocked with blocking buffer for 90 min at 25 °C and then incubated at 4 °C for 18 h with the following primary antibodies: p21 (sc-53,870, 1:1000, Santa Cruz, United States), p53 (sc-100, 1:1000, Santa Cruz, United States), MAFbx (ET7109-25, 1:1000, HuaAn Biotechnology, China), MuRF-1 (A3101, 1:1000, ABclonal, China) and Tubulin (M1305-2, 1:5000, HuaAn Biotechnology, China). After incubation with secondary antibodies (1:5000, Beyotime Inc., China), bands were visible on the membrane with the aid of enhanced chemiluminescence (ECL) reagent. Tubulin and GAPDH were used as loading controls. ImageJ software was utilized for quantitative analysis.

### RNA-seq analysis

Total RNA was extracted and purified, and RNA with an RNA integrity number (RIN) > 7 was used for RNA-seq analysis. After that, the libraries were constructed, and 2 × 150 bp paired-end sequencing (PE150) was performed on an Illumina NovaSeq™ 6000 (LC Biotechnology Co., Ltd. Hangzhou, China) following the protocol. Finally, significant differences in the differentially expressed mRNAs and GO enrichment analyses were performed.

### Statistical analysis

Comparisons between two groups were conducted by unpaired Student’s t tests, and the values are presented as the means ± SDs. Statistical significance was considered when the *p* value was less than 0.05 by SPSS 19.0 statistical software, and GraphPad Prism 9.0 was used to generate graphs.

## Results

### Physiological measurements and respiratory monitoring

**Fig. 1:**
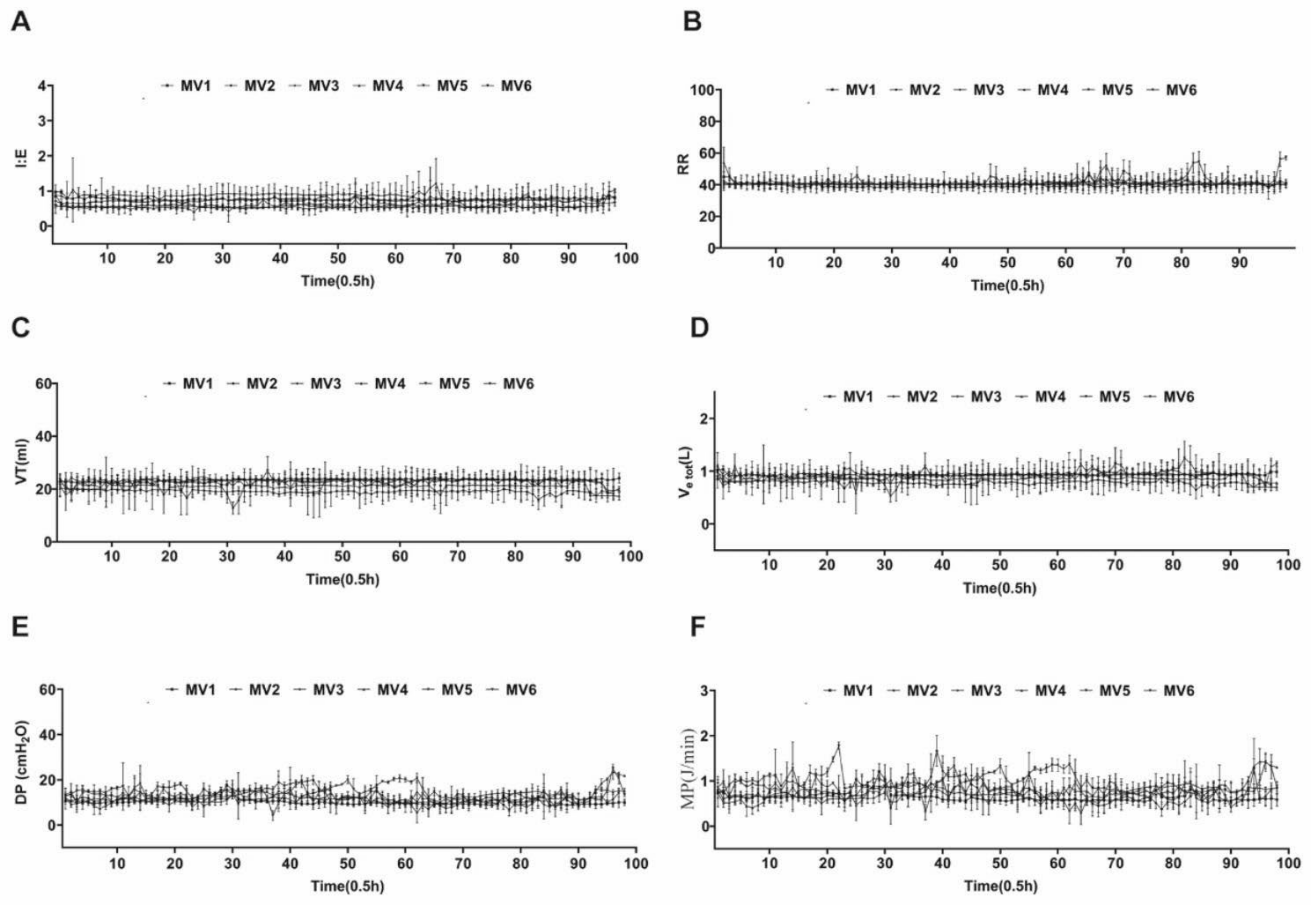
Respiratory parameter monitoring during model establishment. **(A)** Respiratory parameter monitoring of I: E. **(B)** Respiratory parameter monitoring of RR. **(C)** Respiratory parameter monitoring of VT (ml). **(D)** Respiratory parameter monitoring of V_e tot_ (L/min). **(E)** Respiratory parameter monitoring of DP (cmH_2_O). **(F)** Respiratory parameter monitoring of MP (J/min)

The initial body weights between the CON group and the MV group were 2.38 ± 0.03 kg and 2.42 ± 0.03 kg respectively. We monitored the respiratory parameters throughout the process of model establishment (Fig. 1). All the respiratory parameters were monitored with animals anaesthetized under light sedation and myorelaxation-free conditions. During the model establishment, we recorded 109985.33 ± 10159.28 respiratory cycles in each rabbit of the MV group. The inspiration/expiration ratio (I: E) was 0.68 ± 0.12 and respiratory rate was 40.68 ± 0.65. The tidal volume was 21.95 ± 1.67 ml and total minute ventilation (V_e tot_) was 0.89 ± 0.06 L/min. The respiratory driven pressure (DP) of rabbits in the MV group was 12.14 ± 1.2 cmH_2_O and the mechanical power (MP) was 0.77 ± 0.11 J/min. After mechanical ventilated for 48 h, we analyzed the blood gas of each rabbit. As shown in Table 2, the physiological parameters were stably maintained in all animals. The results of the blood gas parameters were not significantly different between the CON group and the MV group.

**Table 2:** Body weight and blood gas analysis in the CON group and the MV group Data are expressed as mean ± SD.

	CON	MV	*P*
Body weight(kg)	2.38 ± 0.03	2.42 ± 0.30	0.802
Lac(mmol/L)	3.17 ± 0.51	3.37 ± 2.01	0.818
PH	7.35 ± 0.03	7.32 ± 0.17	0.720
HCO_3_^−^(mmol/L)	26.87 ± 4.60	22.23 ± 6.75	0.195
PaCO_2_ (mmHg)	48.93 ± 6.22	43.40 ± 12.53	0.355
PaO_2_ (mmHg)	54.87 ± 24.13	50.57 ± 17.90	0.733
FiO2(%)	21.00	21.00	NA
PaO_2_/FiO_2_ (mmHg)	261.27 ± 114.89	240.79 ± 85.23	0.733

### Mechanical ventilation leads to diaphragm injuries and atrophy in healthy rabbits

**Fig. 2:**
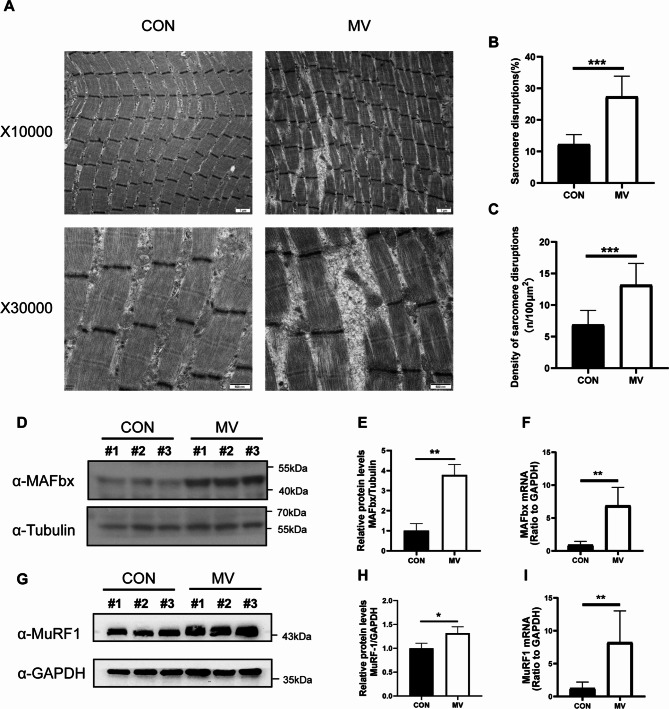
Mechanical ventilation leads to diaphragm injuries and protein degradation. **(A-C)** TEM revealed ultrastructure damage to the diaphragms. Scale bar = 1 μm/500 nm. **(D-E and G-H)** Diaphragm protein lysates of the two groups were detected by Western blotting, and the expression of MAFbx and MuRF1 was quantified. (Before incubated with antibodies, the membranes were clipped according to target proteins molecular weight position on the instruction of the protein marker. Full-length blots are presented in Supplementary Fig. 3). **(F and I)** Diaphragms of the two groups were detected by qPCR to analyse MAFbx and MuRF1 expression. Values are represented as the mean ± SD. n = 6. (*p < 0.05, **p < 0.01, ***p < 0.001)

As shown in Fig. 2A, TEM observations revealed that the ultrastructure of diaphragms in the MV group was altered. Diaphragms in the CON group rabbits showed normal myofibrillar ultrastructure with no anomalies of mitochondrial morphology. However, we observed myofibril fragmentation with a large interfibrillar space and sarcomere disruption on the diaphragms in the MV group. At higher magnification (×30,000), we found that mitochondria presented a disrupted external membrane and blurred internal cristae in the MV group. Morphometric analysis of the diaphragms demonstrated that, compared to those in the CON group, the percentage and density of sarcomere disruption in the MV group were significantly higher (12.28 ± 3.02 and 27.39 ± 6.48, respectively, *p* < 0.001; 6.89 ± 2.25 and 13.20 ± 3.39, respectively, *p* < 0.001) (Fig. 2B-C).

The specific E3 ubiquitin ligases MAFbx and MuRF1 are important regulators of muscle atrophy. Thus, we investigated the mRNA expression of MAFbx and MuRF1 between the CON and MV groups. The qPCR results showed that the mRNA expression of MAFbx and MuRF1 in the MV group was greatly upregulated (0.89 ± 0.55 and 6.87 ± 2.79, respectively, *p* = 0.003; 1.24 ± 0.96 and 8.22 ± 4.80, respectively, *p* = 0.006). Moreover, the western blot results of MAFbx and MuRF1 showed the same trend (1.00 ± 0.35 and 3.78 ± 0.52, respectively, *p* = 0.02; 1.00 ± 0.11 and 1.31 ± 0.13, respectively, *p* = 0.03), indicating diaphragm protein degradation in rabbits after mechanical ventilation (Fig. 2D-I).

### Cellular senescence participates in ventilation-induced diaphragm dysfunction

**Fig. 3:**
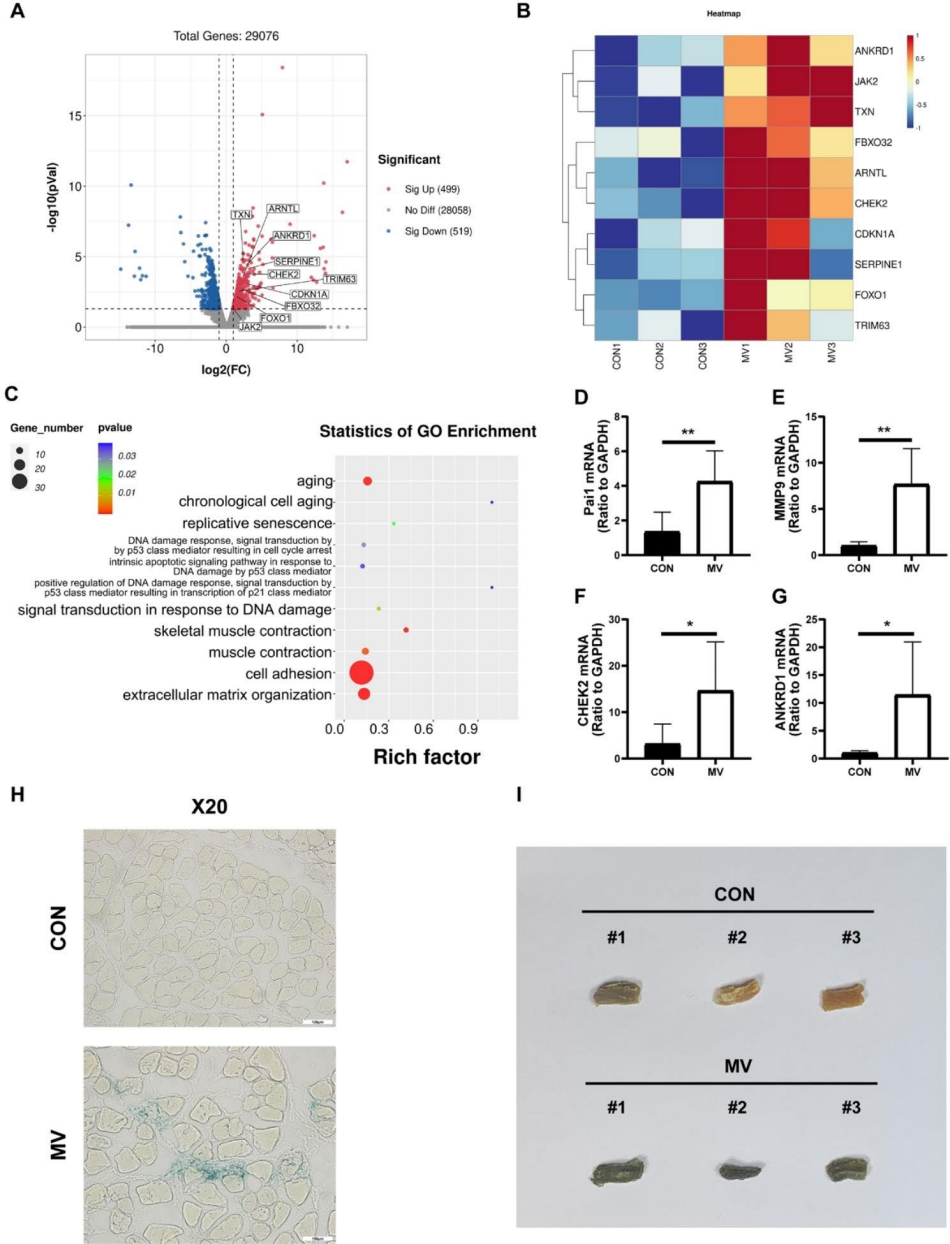
Cellular senescence participates in ventilation-induced diaphragm dysfunction. **(A)** Volcano plot of differential gene expression of the CON group and the MV group (n = 3). **(B)** Heatmap of cellular senescence-related differential gene expression in the CON group and the MV group (n = 3). **(C)** Bubble chart of cellular senescence-related statistics of GO enrichment of the CON group and the MV group (n = 3). **(D-G)** The diaphragm of the two groups was subjected to qPCR to analyse Pai1, MMP9, CHEK2 and ANKRD1 expression (n = 6). **(H-I)** Diaphragmatic tissue sections and holistic pieces of diaphragms from the two groups were subjected to SA-βGal staining. Bar = 100 μm. Values are represented as the mean ± SD. (*p < 0.05, **p < 0.01)

According to the RNA-seq results, we identified a total of 1018 differentially regulated genes among 29,076 genes. Among these, the levels of 499 genes were upregulated and 519 were downregulated in the MV group (Fig. 3A). Interestingly, we found that the MAFbx and MuRF1 genes were upregulated in the MV group, which was in line with our previous results. Moreover, GO analysis of differentially regulated genes showed significant enrichment of genes related to ageing, replicative senescence, signal transduction in response to DNA damage, and positive regulation of DNA damage response-signal transduction by p53 class mediator resulting in transcription of p21 class mediator (Fig. 3C). These findings suggest that senescence is potentially involved in VIDD. By further probing our data, we found some differentially regulated genes associated with cellular senescence, as shown in Fig. 3A and B. Additionally, the mRNA expression of senescence-related genes, including Pai1, MMP9, CHEK2 and ANKRD1, was upregulated in the MV group (Pai1: 1.35 ± 1.13 and 4.25 ± 1.78, *p* = 0.009; MMP9: 1.03 ± 0.40 and 7.67 ± 3.88, *p* = 0.008; CHEK2: 3.16 ± 4.30 and 14.64 ± 10.52, *p* = 0.033; ANKRD1: 1.05 ± 0.36 and 11.44 ± 9.51, *p* = 0.023) (Fig. 3D-G). These results indicate that cellular senescence plays a potent role in the pathogenesis of VIDD.

Senescence has many heterogeneous phenotypes, making it complicated to describe in living animals. Thus, multiple lines of evidence are required to better identify the senescent state [26]. We next utilized SA-βGal staining to assess whether mechanical ventilation indeed induced senescence in the diaphragm. SA‐βGal staining is the most widely utilized senescence marker to pinpoint senescent cells in vivo [28, 29]. As shown in Fig. 3H and I, the transverse cross-sections of diaphragm myofibrils in the MV group showed positive staining of SA‐βGal, while positive staining was hardly found in the CON group. Whole-piece staining of the diaphragm also showed more intensive staining of SA‐βGal in the MV group than in the CON group. Taken together, these findings support the conclusion that senescence contributes to VIDD.

### Cellular senescence causes ventilation-induced diaphragm dysfunction via elevation of the p53-p21 axis

**Fig. 4:**
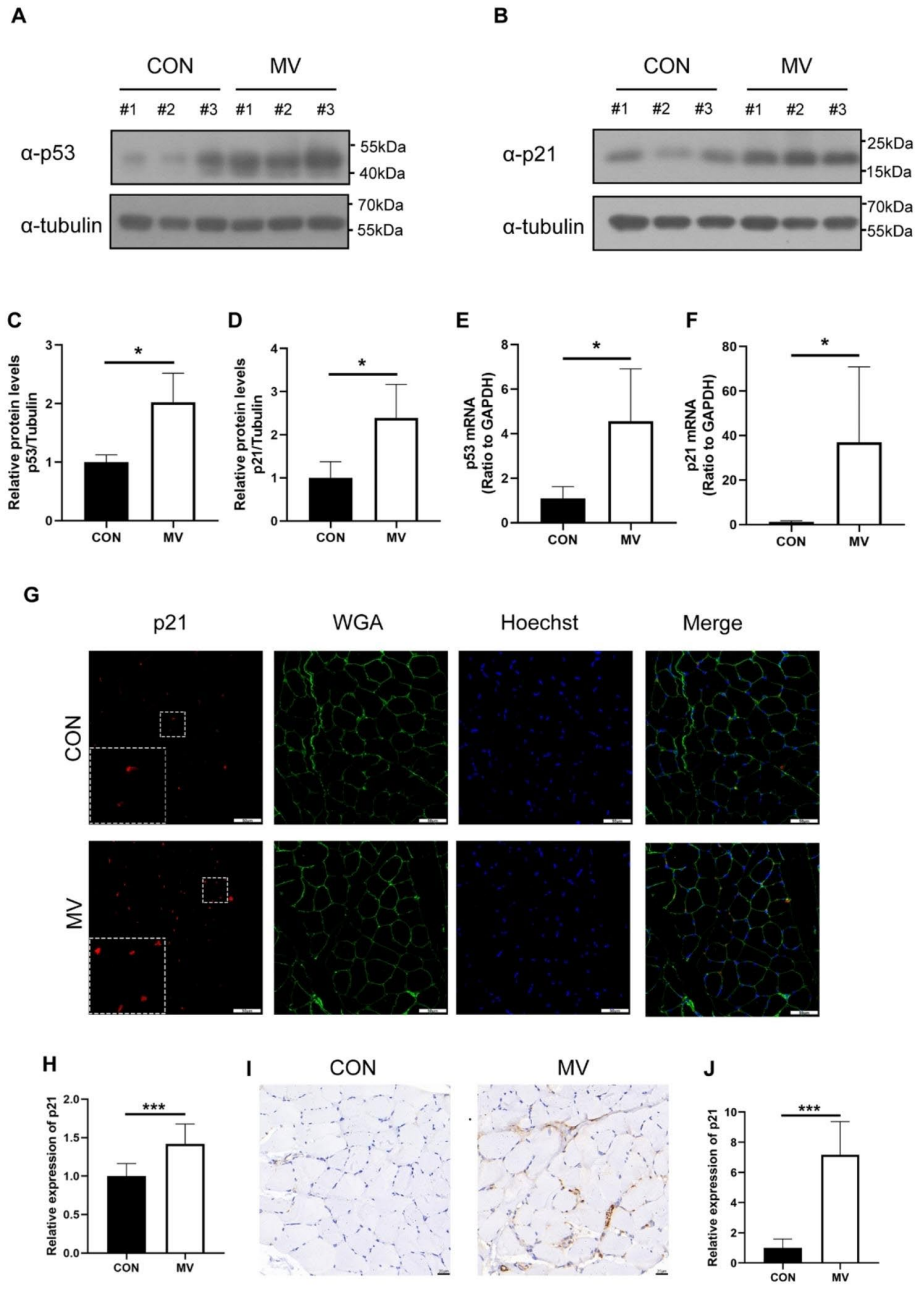
Cellular senescence causes ventilation-induced diaphragm dysfunction via elevation of the p53-p21 axis. **(A-D)** Diaphragm protein lysates of the two groups were detected by western blot analysis, and the expression of p53 and p21 was quantified. (Before incubated with antibodies, the membranes were clipped according to target proteins molecular weight position on the instruction of the protein marker. Full-length blots are presented in Supplementary Fig. 3). **(E and F)** The diaphragm of the two groups was detected by qPCR to analyse the expression of p53 and p21. **(G and H)** Diaphragmatic tissue sections were subjected to immunofluorescence staining to analyse the expression of p21. Red represents p21; Green represents WGA; Blue represents Hoechst. Scale bar = 50 μm. **(I and J)** Diaphragmatic tissue sections were subjected to immunohistochemistry staining to analyse the expression of p21. Brown represents p21. Scale bar = 20 μm. Values are represented as the mean ± SD. n = 6. (*p < 0.05, **p < 0.01, ***p < 0.001)

As we have mentioned above, p53-p21 axis-related biological processes were detected in the GO analysis of differentially regulated genes between the CON group and MV group. Additionally, we noticed that the p21 gene was upregulated in the MV group. Thus, we detected the expression levels of p53 and p21 in the diaphragm between these two groups. Compared to those in the CON group, the mRNA expression levels of p53 and p21 were greatly regulated in the MV group (1.10 ± 0.52 and 4.55 ± 2.36, respectively, *p* = 0.02; 1.16 ± 0.56 and 36.87 ± 34.02, respectively, *p* = 0.05) (Fig. 4E and F). The same trend was observed in the western blot results, which showed that the expression of p53 and p21 was greatly upregulated in the MV group (1.00 ± 0.12 and 2.02 ± 0.50, respectively, *p* = 0.03; 1.00 ± 0.37 and 2.39 ± 0.78, respectively, *p* = 0.05) (Fig. 4A-D). We then carried out IF and IHC to detect the p21 expression level in the transverse cross section of diaphragm myofibrils. In line with our previous findings, the p21-positive staining in the MV group was significantly more intense than that in the CON group (1.00 ± 0.16 and 1.42 ± 0.26, respectively, *p <* 0.001; 1.00 ± 0.59 and 7.17 ± 2.20) (Fig. 4G -J).

## Discussion

Consistent with previous studies, the current study found that spontaneous breath was preserved under ACV mode in rabbits, and the fluctuation of respiratory parameters reflected the autonomic respiration drive (Fig. 1). However, we revealed that mechanical ventilation in the assit/ control (A/C) mode for 48 h still significantly induced ultrastructural damage and atrophy of the diaphragm in healthy rabbits (Fig. 2). We found disrupted myofibrils with a large interfibrillar space and damaged sarcomeres in the diaphragm after ventilation. Structurally injured myofibrils are associated with impaired force-generating capacity [7]. At higher magnification, swollen mitochondria with a disrupted external membrane could be observed. In addition, upregulation of MAFbx and MuRF1 was also detected, indicating activation of the UPS and enhanced catabolic activity. Increased protein ubiquitination is one of the central pathogenic events underlying VIDD [30, 31]. Moreover, we found that cellular senescence induced by p53 signalling pathways participates in the mechanisms of VIDD in rabbits.

Skeletal muscle is an important organ in senescence, and activation of cellular senescence plays a potent role in muscle dysfunction [20]. It is well established that senescence can lead to muscle mass loss and frailty [32], and loss of muscle function is one of the most prevalent senescence-related disorders. However, recent researchers have indicated that injury-induced senescence can promote muscle reprogramming and that the SASP is beneficial in tissue repair [21, 33]. p53 is the main regulator of p21, which increases transcription of p21 [34]. The activation of p21 inhibits CDK2-mediated RB phosphorylation to promote cell cycle arrest [26], which is an important pathway for cellular senescence to be activated. Studies indicate that enhanced and properly regulated p53 activity prevents cellular stress and promotes longevity. However, when the dose of p53 exceeds the threshold, the function of p53 transforms into driving cell death [35]. Notably, persistent p53-dependent senescence plays a detrimental role in muscle repair [23]. It has been reported that inhibition of Hsp90β can promote p53‐dependent senescence to impair the process of muscle regeneration [36]. Likewise, among muscle atrophy in the diaphragm induced by cigarette smoke exposure in rats, there is upregulation of p53 and p21 expression [27]. In hindlimb-immobilized mice, there were increases in ATF4, p53 and p21 protein levels. Moreover, knockout of ATF4/p53 or inhibition of p21 moderates muscle injury [37]. In current study, the results of RNA-seq revealed that the expression of senescence-related genes was significantly upregulated in the MV group, suggesting that senescence is potentially involved in VIDD. Further studies noticed that p53-p21 axis was elevated, which revealed a novel molecular event contributing to the development of VIDD in ACV mode for 48 h.

Based on the present understanding of VIDD, many studies have attempted to alleviate the injury induced by mechanical ventilation. In terms of molecular mechanisms, it has been suggested that treatment with angiotensin 1–7 can activate the nonclassical RAS signalling pathway to protect against VIDD by diminishing oxidative stress and protease activation [38]. Increasing amounts of evidence have revealed that hydrogen sulfide (H_2_S) exerts a protective effect against oxidative damage, proteolysis, and mitochondrial injury induced by VIDD [39]. The chaperone coinducer BGP-15 may help reduce VIDD by downregulating the PARP pathway and HSP72 expression [40]. In terms of ventilation strategy, our previous research has demonstrated that high-level pressure support ventilation alleviates VIDD in rabbits [41]. It has also been shown that a neurally adjusted ventilatory assist mode improves interactions between the patient and ventilator, which could prevent diaphragmatic sarcomere injury and myofibrotic cell apoptosis [42]. Our present research reveals p53-dependent cellular senescence as a novel mechanism of VIDD. However, more efforts are needed to transform the findings into a mature clinical treatment to alleviate VIDD.

There were several limitations in this study. First, we were unable to measure muscle contractile properties to best describe the functional alteration of the diaphragm. In addition, although we monitored the airway peak pressure throughout to assess the stability of model establishment, diaphragmatic electromyography may have reflected the unloading of the diaphragm more directly. Thus, we must improve the experimental design in the future. Further studies should be carried out to explore the upstream mechanisms of senescence-mediated VIDD and to explore relevant strategies to prevent VIDD.

## Conclusions

Upon establishing a mechanical ventilation model in rabbits, we demonstrated that mechanical ventilation in ACV mode for 48 h can still significantly induce ultrastructural damage and atrophy of the diaphragm. Moreover, p53-dependent senescence might play a role in mechanical ventilation-induced dysfunction. These findings might provide novel therapeutic targets for VIDD.

### Electronic supplementary material

Below is the link to the electronic supplementary material.

Supplementary Material 1

Supplementary Material 2

Supplementary Material 3

Supplementary Material 4

Supplementary Material 5

Supplementary Material 6

## Data Availability

The datasets generated during the current study are available in the Gene Expression Omnibus (GEO) repository, and are accessible through GEO Series accession number GSEGSE228739 (https://www.ncbi.nlm.nih.gov/geo/query/acc.cgi?acc=GSE228739) after 05/Apr/2026.

## Abbreviations

ACV: Assisted-controlled ventilation
CDKi: Cyclin-dependent kinase inhibitors
CMV: Controlled mechanical ventilation
ECL: Enhanced chemiluminescence
IF: Immunofluorescence
IHC: Immunohistochemistry
MOS: Mitochondrial oxidative stress
SASP: Senescence-associated secretory phenotype
TEM: Transmission electron microscope
UPS: Ubiquitin proteasome system
VIDD: Ventilation-induced diaphragm dysfunction
