# Supplementary figures and images for: Cellular senescence contributes to mechanical ventilation-induced diaphragm dysfunction by upregulating p53 signalling pathways
Source: BMC Pulm Med. 2023 Dec 14;23:509. doi: 10.1186/s12890-023-02662-7 (PMC10722656; doi:10.1186/s12890-023-02662-7)

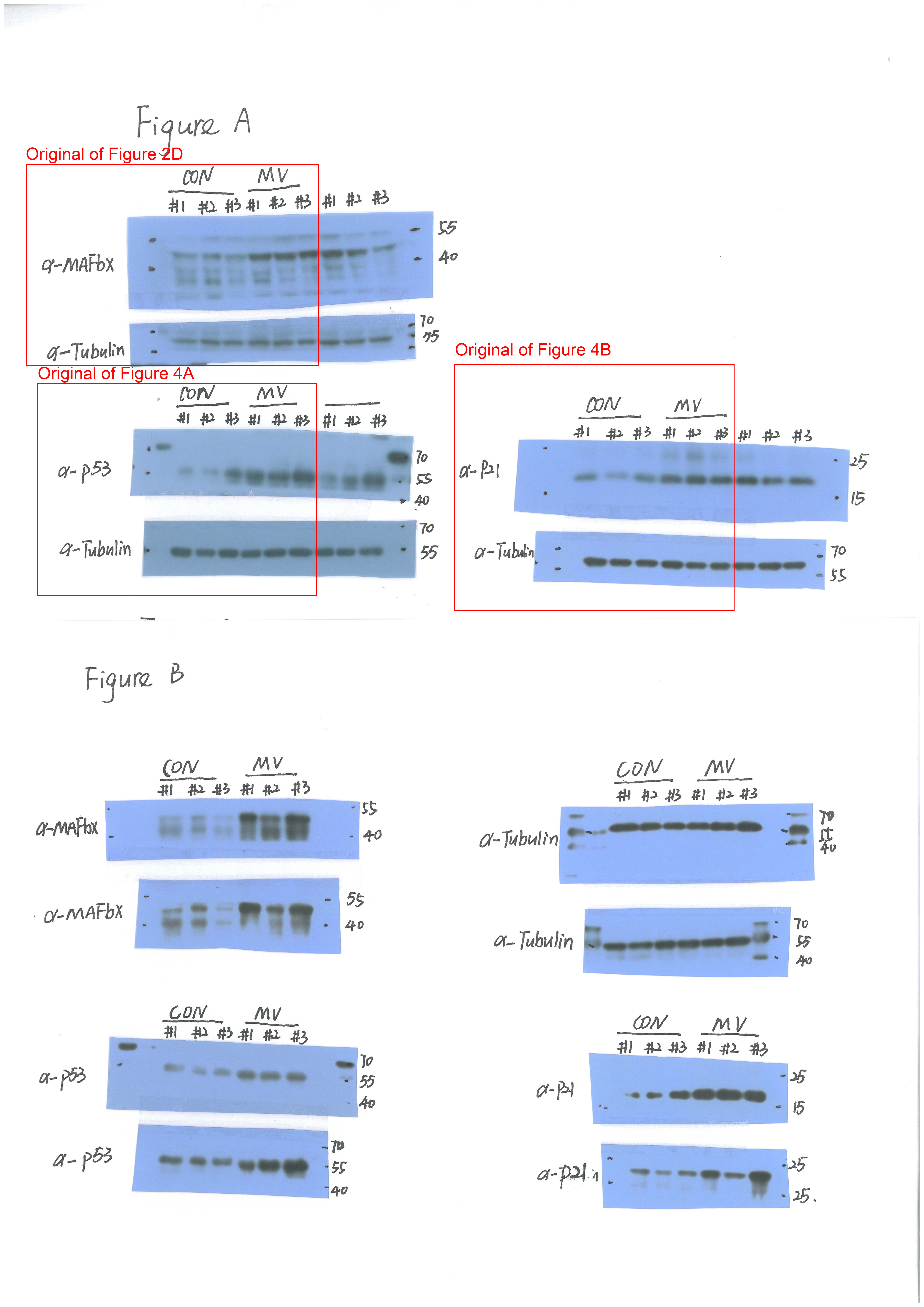

Supplement: Supplementary file 1 — Supplementary Material 1 [file 12890_2023_2662_MOESM1_ESM.tif]

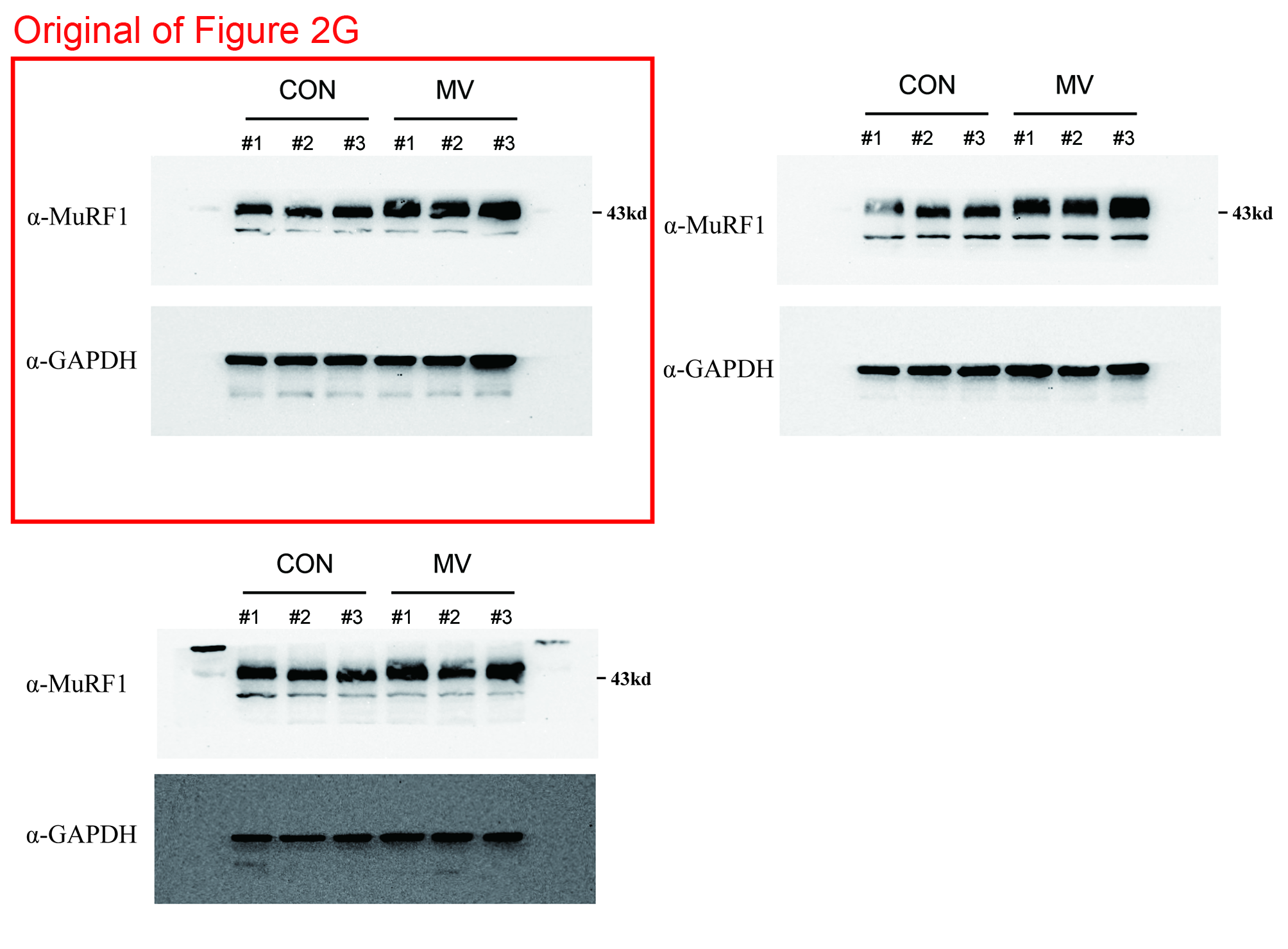

Supplement: Supplementary file 2 — Supplementary Material 2 [file 12890_2023_2662_MOESM2_ESM.tif]

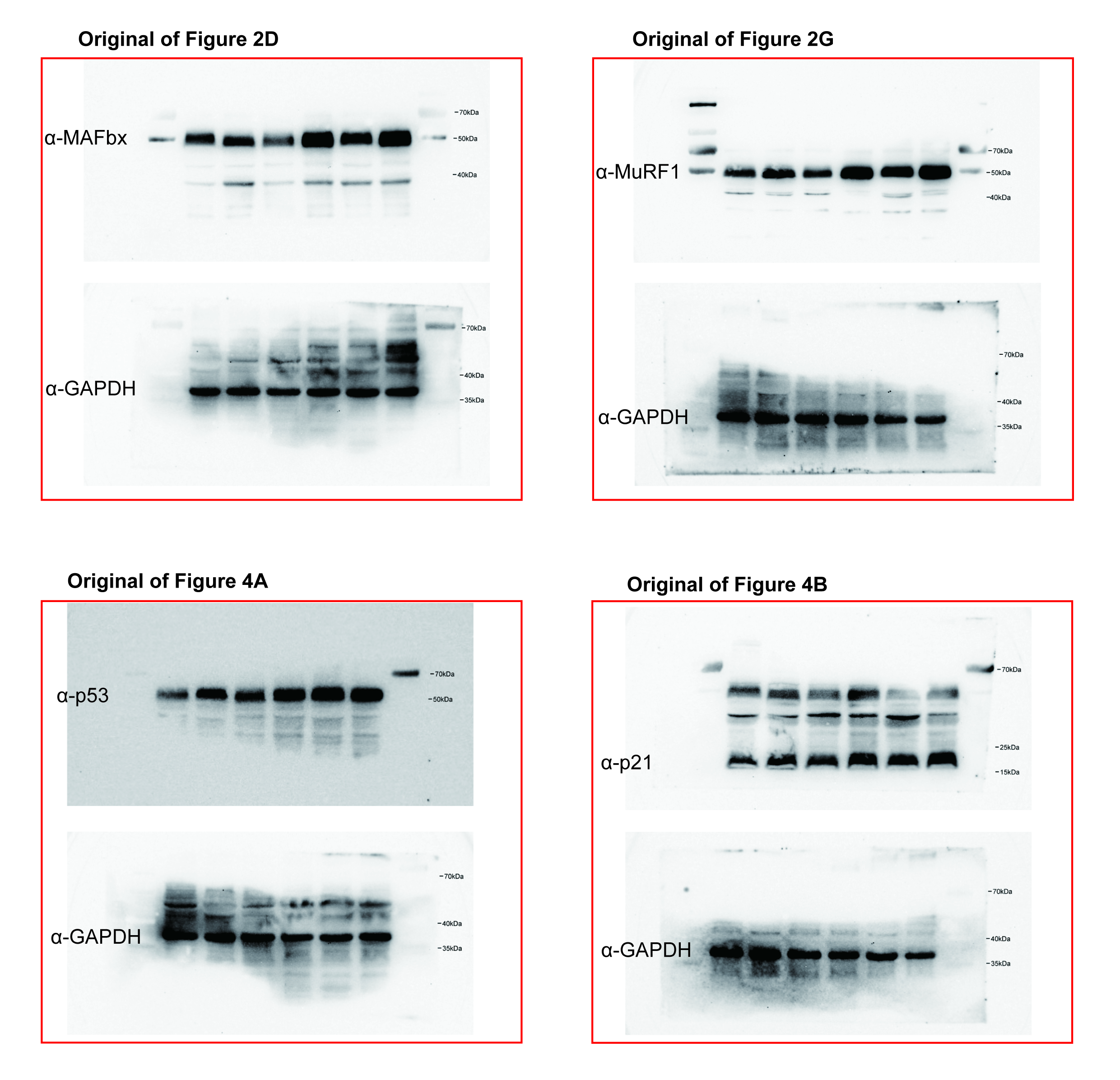

Supplement: Supplementary file 3 — Supplementary Material 3 [file 12890_2023_2662_MOESM3_ESM.tif]
