# Supplementary material for: Cellular senescence contributes to mechanical ventilation-induced diaphragm dysfunction by upregulating p53 signalling pathways
Source: BMC Pulm Med. 2023 Dec 14;23:509. doi: 10.1186/s12890-023-02662-7 (PMC10722656; doi:10.1186/s12890-023-02662-7)
Supplement: Supplementary file 4 — Supplementary Material 4 [file 12890_2023_2662_MOESM4_ESM.docx]

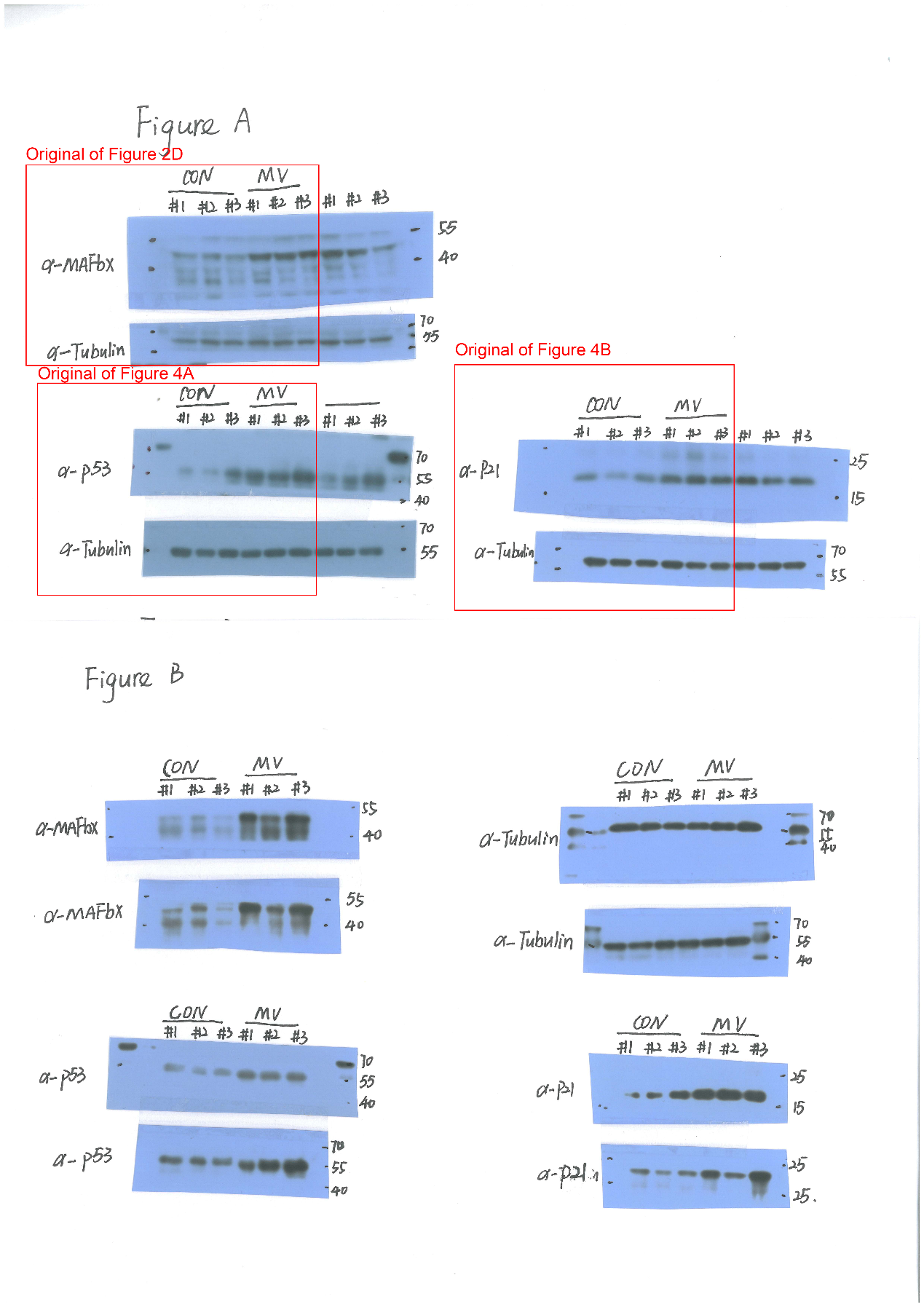


The original, full-length blot images was provided. Please allow me to make some explanations to the supplemental Figure1.

As shown in Figure A, blot images of the first three lanes were from the CON group, blot images of the next three lanes were from the MV group, and blot images of the last three lanes were from samples for other researches.

We repeated the experiment to verify the accuracy of the experimental results. Blot images were shown in Figure B. Blot images of the first three lanes were from the CON group, and blot images of the last three lanes were from the MV group. Out of concern for unifying the sample and better exhibiting the trend of the results, we choose the bolt images in Figure A.

Adjacent protein markers of target protein have been mentioned in the legend.
