# Supplementary material for: Cellular senescence contributes to mechanical ventilation-induced diaphragm dysfunction by upregulating p53 signalling pathways
Source: BMC Pulm Med. 2023 Dec 14;23:509. doi: 10.1186/s12890-023-02662-7 (PMC10722656; doi:10.1186/s12890-023-02662-7)
Supplement: Supplementary file 5 — Supplementary Material 5 [file 12890_2023_2662_MOESM5_ESM.docx]

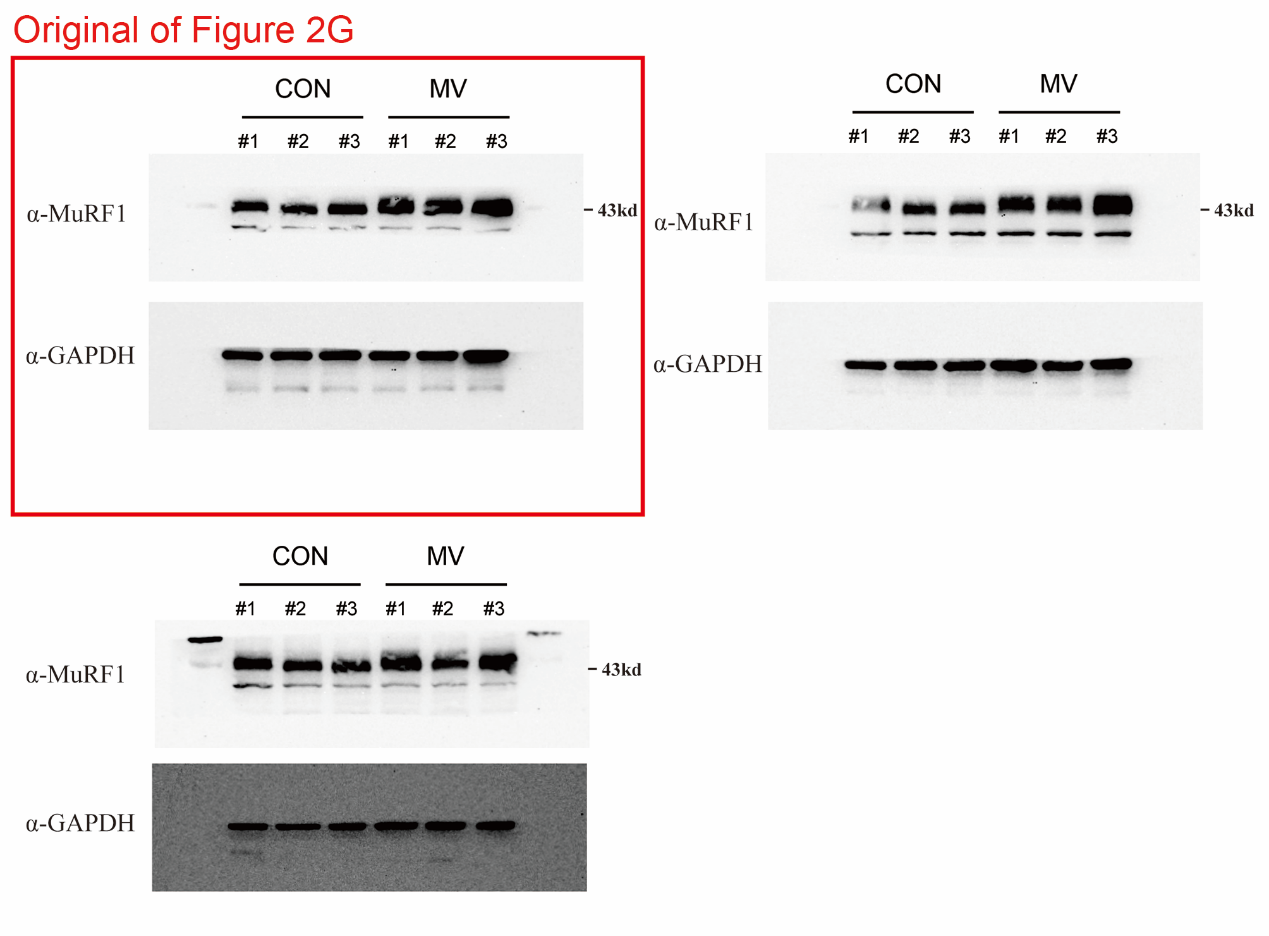


Our lab equipment was updated，therefore，images of western blot during revison were exposed by Gel imaging system (ChemiDoc XRS+, Bio-RAD, USA). It may cause the difference in image representation compared to previous images.

Blot images of the first three lanes were from the CON group, and blot images of the last three lanes were from the MV group. Adjacent protein markers of target protein have been mentioned in the legend.
