# Supplementary material for: Cellular senescence contributes to mechanical ventilation-induced diaphragm dysfunction by upregulating p53 signalling pathways
Source: BMC Pulm Med. 2023 Dec 14;23:509. doi: 10.1186/s12890-023-02662-7 (PMC10722656; doi:10.1186/s12890-023-02662-7)
Supplement: Supplementary file 6 — Supplementary Material 6 [file 12890_2023_2662_MOESM6_ESM.docx]

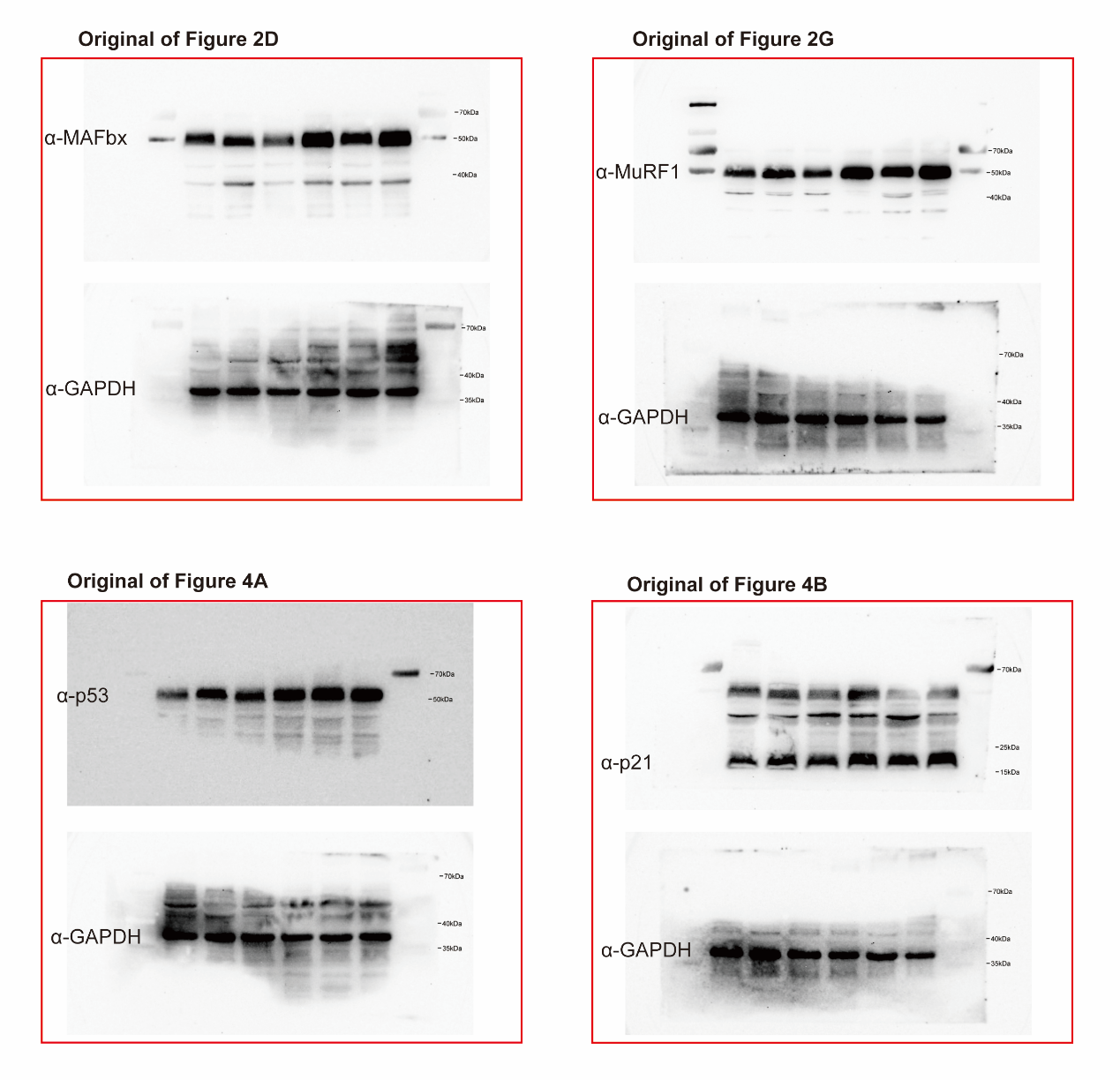


Fuller-length, original, unprocessed blotswere provided in supplementary Figure 3.

Blot images of the first three lanes were from the CON group, and blot images of the last three lanes were from the MV group. Adjacent protein markers of target protein have been mentioned in the legend.
